# Cybervictimization and non-suicidal self-injury among Chinese adolescents: The effect of depression and school connectedness

**DOI:** 10.3389/fpubh.2023.1091959

**Published:** 2023-03-08

**Authors:** Sui Liu, Wanchun Wu, Hongyu Zou, Yanrong Chen, Liling Xu, Wei Zhang, Chenfu Yu, Shuangju Zhen

**Affiliations:** ^1^School of Psychology, South China Normal University, Guangzhou, China; ^2^School of Foreign Studies, South China Normal University, Guangzhou, China; ^3^School of Education, Guangzhou University, Guangzhou, China

**Keywords:** cybervictimization, depression, school connectedness, adolescent, non-suicidal self-injury

## Abstract

Cybervictimization has been shown in many studies to be a risk factor for adolescent non-suicidal self-injurious behavior (NSSI). In this study we tested the roles of depression and school connectedness in this association. The Integrative Model of NSSI, Emotion Regulation and Interpersonal Relationship Model of NSSI, and Integrative Model of Social Media and Suicide provided the conceptual framework for the study. A sample of 1106 adolescents (*M*_*age*_ = 13.17; *SD* = 0.69; 51.78% girls) completed anonymous questionnaires in their classrooms. The results of structural equation modeling showed that the positive association between cybervictimization and adolescent NSSI was mediated by depression. Moreover, this indirect link was stronger for adolescents with low vs. high school connectedness. The results have implications for intervention programs aimed at reducing NSSI among adolescents.

## 1. Introduction

Non-suicidal self-injury (NSSI) is a deliberate behavior enacted for the purpose of self-harm but not suicide, the behavior is considered unacceptable in the societal or cultural context in which it occurs, the most common forms of NSSI include cutting, burning, and scratching (1, 2). NSSI often develops in adolescence (3), and in recent years, adolescent NSSI has become a major public health problem (4). About 17.2% of adolescents in the world have engaged in NSSI (5), and its prevalence is increasing every year (6). In a meta-analysis involving 597,548 adolescents, the lifetime prevalence (7) and another two research showed that 12-month prevalence of NSSI were 16.9 and 14–39%, respectively (8, 9). A nationwide study of 150,000 adolescents in China, where the current study was conducted, revealed that ~22.37% had engaged in NSSI (8).

Although NSSI is not enacted for the purpose of suicide, it is nevertheless strongly associated with suicidal behavior, suicide attempts, and suicide (10, 11). In one study, having shown NSSI more than seven times increased a person's risk of suicide (12). Adolescent suicidal behavior is significantly predicted by NSSI (13, 14), and in many situations, NSSI is more predictive of suicide attempts than suicidal behavior (15).

In the last 10 years, researchers have concentrated on the mechanisms (1, 16, 17), risk factors (18, 19), and biological markers of NSSI, (20), including the HPA axis (21) and exosomes (22), and have achieved significant advances in understanding NSSI. However, with the development of science and technology, adolescents' life fields have now moved from the physical world to the virtual online world. Adolescents can communicate with people on the Internet anytime and anywhere, and they may also have friction and experience cybervictimization. Interpersonal risk factors such as cybervictimization were also common in adolescent, in one study, with ~30.7% of participants having experienced cyberbullying. Boys were more likely to be victims of cyberbullying than girls. The findings also indicate that cybervictimization is a significant risk factor for self-injury among adolescents (23).

Cybervictimization refers to someone were bullied by other people who using digital technology, including repeatedly intimidating, provoking, or humiliating others through social media, online communication platforms, gaming platforms, and SMS on mobile phones (24). This problem has become a serious social problem that can affect adolescents' mental health (25). Cybervictimization was associated with negative emotions in adolescents and is positively connected with a wide range of psychiatric issues (24). A meta-analysis which comprising 11 cross-sectional studies has revealed that cybervictimization may increase the risk of NSSI (26). A longitudinal study also indicated that cybervictimization was an important positive predictor of NSSI in adolescents (27). Admittedly, the association between the cybervictimization and adolescent NSSI was exists. However, the mechanism of this association is unclear.

Cybervictimization is not always associated with NSSI. Among youth who do engage in NSSI, the self-injurious behavior typically does not occur immediately; the adolescent goes through an emotional process that includes struggles, retaliation, rage, and sadness before eventually expressing their repressed feelings through NSSI (1, 27). Recent research has demonstrated that adolescents who experience cybervictimization are more likely to experience negative feelings, which can lead to a variety of psychological issues like depression and anxiety (28). For instance, a prospective study of 2,480 adolescents showed that depression was positively predicted by peer cybervictimization (29). Similar findings were obtained from a longitudinal study of 559 youth in grades 6–12 (30). Cybervictimization at Time 1 and Time 2 significantly predicted depression and anxiety in adolescents at Time 2 and Time 3.

However, there are also longitudinal studies showing that cybervictimization cannot significantly predict the occurrence of depression in adolescents (31). The main reason for these mixed results may be the existence of mediating variables in the association between cybervictimization and adolescent NSSI. Additionally, according to several conceptual models, including Nock's integration model and the emotional regulation model, NSSI is frequently used as a coping mechanism in response to negative emotion (1, 32), and unpleasant emotions like depression and anxiety can significantly predict the occurrence of NSSI (33). A longitudinal study of 6,995 Chinese adolescents over the time period of 2 years revealed that depression at Time 1 strongly predicted NSSI at Time 3 (34). Although previous studies have demonstrated relationships among cybervictimization, depression, and NSSI, no studie have examined the mediating role of depression in the association between cybervictimization and NSSI. Therefore, we hypothesized that depression mediates the relationship between cybervictimization and NSSI.

Individual differences might explain why different people show different psychological responses to the same negative event. For example, resilience has been shown to weaken the association between negative life events and depression (35, 36). Another individual differences were socail support and school connectedness (37, 38). School connectedness refers to students' sense of attachment and belonging to the school and its environment, and is reflected in students' emotion, behavior and cognition (39). During adolescence, youth spend more time in school activities, and the social support offered by schools increases opportunities for the positive development of adolescent mental health (40, 41).

From the perspective of the occurrence of non-suicidal self-injury and protective factors, according to the theory of social support in NSSI, people exhibit less NSSI when they have outside support (42). Thus, the association between cybervictimization and NSSI may be weaker when there is high school connectedness as a form of social support. No studies to date have tested the association between cybervictimization and NSSI, and thus moderators of this association have also not been tested. Indirect evidence of a possible link comes from research showing that school connectedness may moderate the association between bullying and maladjustment (e.g., impulsive behavior, depression, and anxiety) (43–45). Additionally, Wang et al. demonstrated that the relation between cybervictimization and suicidal ideation was only statistically significant when there was low school connectedness (46).

**Figure 1:**
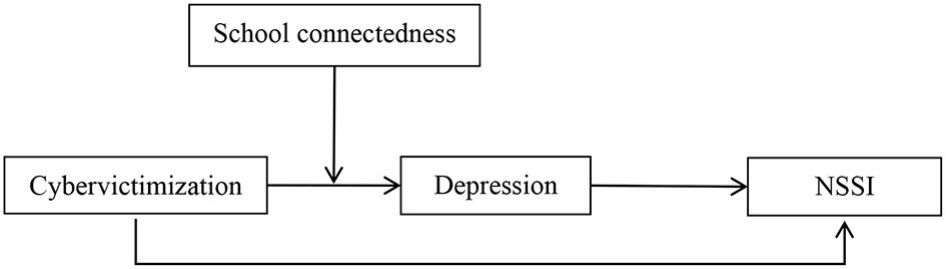
The proposed mediated moderation model. NSSI, non-suicidal self-injurious behavior.

Figure 1 shows the hypothesized moderated mediation model. School connectedness was tested as a moderator of the mediated process by which cybervictimization is associated with NSSI by way of depression. Specifically, we tested whether school connectedness moderates the first link in this mediated path, namely the association between cybervictimization and depression. The results of these analyses have implications for identifying a mechanism by which NSSI develops, and for developing interventions based on school connectedness as a protective factor. In this manuscript, we hope to further investigate the relationship between cyber victimization and NSSI and its internal occurrence mechanism by exploring the relationship between these four variables, as well as to propose protective factors for the problem of non-suicidal self-injury in adolescents from the perspective of interpersonal relationships and social support. To further guide future research and provide guidance on the prevention and intervention of NSSI in school adolescents.

***Hypothesis 1*. **Cybervictimization will be positively associated with adolescent NSSI.
***Hypothesis 2*. **Depression will mediate the association between cybervictimization and adolescent NSSI.
***Hypothesis 3*. **The indirect relationship between cybervictimization and adolescent NSSI *via* depression will be moderated by school connectedness. Specifically, school connectedness will weaken the link between cybervictimization and depression.

## 2. Methods

### 2.1. Participants and procedure

Participants were recruited from three junior middle schools in Guangdong province, southern China, through stratified and random cluster sampling. A total of 1,006 adolescents (51.78% girls, *n* = 521) ranging in age from 12 to 15 (*M*_age_ = 13.16, *SD* = 0.67) completed questionnaires in their classrooms. There were 556 seventh graders and 450 eighth graders. Regarding the demographics of the sample, 48.63% of participants' fathers and 54.07% of their mothers had less than a high school education. The students lived in rural (44.51%) and urban (55.49%) areas.

### 2.2. Questionnaire survey

#### 2.2.1. Cybervictimization

Cybervictimization was assessed using the Chinese version (47) of the Cybervictimization Scale (Erdu-Baker and Kavsut et al.). Participants were asked to use a four-point Likert scale, from 1 (never) to 4 (more than 5 times), to report how often they had experienced 18 forms of cybervictimization in the previous 6 months. An example item is, “Someone propagated rumors about me online.” The frequency of cybervictimization increases with the Cybervictimization Scale score. The scale's Cronbach's alpha in this study was 0.82, which indicates strong internal consistency.

#### 2.2.2. Depression

Depression was assessed using The Center for Epidemiologic Studies Depression Scale updated by Liu et al. (48, 49). On a four-point Likert scale, from 1 (never) to 4 (always), participants stated how frequently they had experienced symptoms of depression throughout the previous week (for example, “I felt like everything I did was an effort”). A higher score indicates higher depressive symptoms. Cronbach's alpha in this study was 0.88, indicating strong internal consistency.

#### 2.2.3. School connectedness

The Emotional Engagement Sub-scale of the School Engagement Scale, developed by Wang et al. (50), assesses adolescents' sense of school connectedness. The measure has eight statements (for example, “In general, I feel like a true member of this school”), and respondents were asked to rate their thoughts on a five-point Likert scale from 1 (strongly opposed) to 5 (strongly in favor). The average rating was calculated as the final score, and a higher score on the scale denotes a higher level of school connectedness. The scale's internal consistency in this study was good, as indicated by its Cronbach's alpha of 0.76.

#### 2.2.4. NSSI

NSSI was measured with the Non-Suicidal Self-Injury scale (NSSI) (51), which includes seven items referring to behaviors such as self-cutting, burning and biting. Participants were asked to report whether they had engaged in each behaviors without suicidal intent in the past 6 months. Items were rated on a four-point scale (1 = never, 2 = once or twice, 3 = three to five times, 4 = six or more times). The reliability and validity of the scale are good. A higher score indicates higher NSSI situation. This scale's internal consistency in this study was good, as indicated by its Cronbach's alpha of 0.71.

### 2.3. Control variables

Gender (male/female), age, and score on the measure of sensation seeking were used as covariates in the analyses. Sensation seeking was measured using the Chinese version of the sensation seeking scale. These variables have all been shown to be associated with risk of NSSI (52, 53).

### 2.4. Procedure

This research was approved by the Academic Ethics Review Committee of the School of Education, Guangzhou University. Informed consent was obtained from teachers, parents, and participating adolescents prior to information collection. Participating adolescents spent ~30 min in their regular classroom completing a series of self-report questionnaires. Data were collected by trained psychology teachers or graduate psychology students. To encourage honest responses, participants were informed that their responses would be kept strictly confidential and that their participation was voluntary and that they could refuse to participate in the study at any time.

### 2.5. Statistical analyses

SPSS 25.0 version was used to generate descriptive statistics and correlations. Mplus 8.1 was used to conduct structural equation modeling using maximum likelihood estimation and bias-corrected percentile bootstrapping with 5,000 replications. The analyses tested whether the association between cybervictimization and NSSI was mediated by symptoms of depression. They also tested whether school connectedness moderated (weakened) this indirect association. Specifically, school connectedness was tested as a moderator of the link between cybervictimization and depression; moderation in any link constitutes moderation of the whole mediation model. We controlled for gender, age, and sensation seeking as predictors in regression equations as part of structural equation modeling.

## 3. Results

### 3.1. Preliminary analyses

The means, standard deviations, and correlation coefficients for all variables are displayed in Table 1. Cybervictimization, depression, and NSSI were significantly inter-correlated (*p* < 0.001). Moreover, school connectedness was negatively correlated with depression (*p* < 0.001).

**Table 1:** Descriptive statistics and correlations for all variables.

**Variable**	**1**	**2**	**3**	**4**
1. Cybervictimization	1.00			
2. School connectedness	0.01	1.00		
3. Depression	0.29^***^	−0.14^***^	1.00	
4. NSSI	0.24^***^	−0.03	0.38^***^	1.00
Mean	1.13	3.70	1.71	1.09
SD	0.20	0.90	0.48	0.29

^***^p < 0.001. NSSI, non-suicidal self-injurious behavior.

### 3.2. Testing for mediation effect of depression

**Figure 2:**
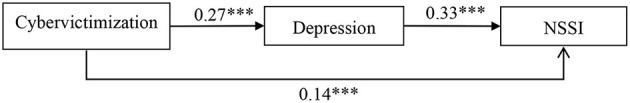
Model of the mediating role of depression in the association between cybervictimization and NSSI. NSSI, non-suicidal self-injurious behavior. ***p < 0.001.

The mediation model represented in Figure 2 showed an excellent fit to the data: χ^2^/*df* = 0.19, CFI = 1.00, RMSEA = 0.00, and SRMR = 0.01. The mediation effect is considered significant if the confidence interval (*CI*) does not include 0. After controlling for gender, age, and sensation seeking, cybervictimization positively predicted depression (β = 0.27, *SE* = 0.03, *t* = 9.00, *p* < 0.001, 95% *CI* [0.21, 0.33]), and depression positively predicted NSSI (β = 0.33, SE = 0.03, *t* = 10.71, *p* < 0.001, 95% CI [0.27, 0.39]). Moreover, the residual effect of cybervictimization on NSSI was significant (β = 0.14, SE = 0.03, *t* = 4.59, *p* < 0.001, 95% CI [0.08, 0.20]). Bootstrapping analyses indicated that depression significantly mediated the relation between cybervictimization and adolescent NSSI (indirect effect = 0.09, SE = 0.02, 95% CI [0.06, 0.14]).

### 3.3. Testing for moderated mediation

**Figure 3:**
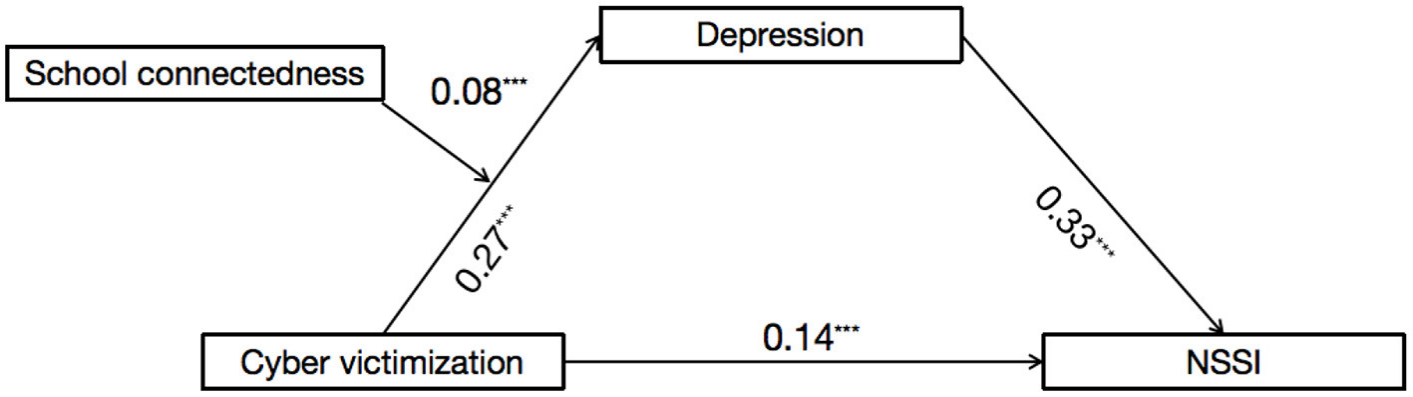
Model of the moderating role of school connectedness on the indirect relationship between cybervictimization and NSSI. NSSI, non-suicidal self-injurious behavior. ***p < 0.001.

The moderated mediation model represented in Figure 3 showed a good fit to the data: χ^2^/*df* = 1.96, CFI = 0.96, RMSEA = 0.04, and SRMR = 0.03. The moderation effect is considered significant if the confidence interval does not include 0. The results showed that school connectedness moderated the association between cybervictimization and depression (β = 0.08, *SE* = 0.03, *t* = 2.64, *p* < 0.01, 95% CI [0.02, 0.14]). Moreover, cybervictimization had a significant positive association with depression (β = 0.27, *SE* = 0.03, *t* = 9.10, *p* < 0.001, 95% CI [0.21, 0.33]) and NSSI (β = 0.14, *SE* = 0.03, *t* = 4.59, *p* < 0.001, 95% CI [0.08, 0.20]). The predictive effect of depression on NSSI was significant (β = 0.33, *SE* = 0.03, *t* = 10.71, *p* < 0.001, 95% CI [0.27, 0.39]).

**Figure 4:**
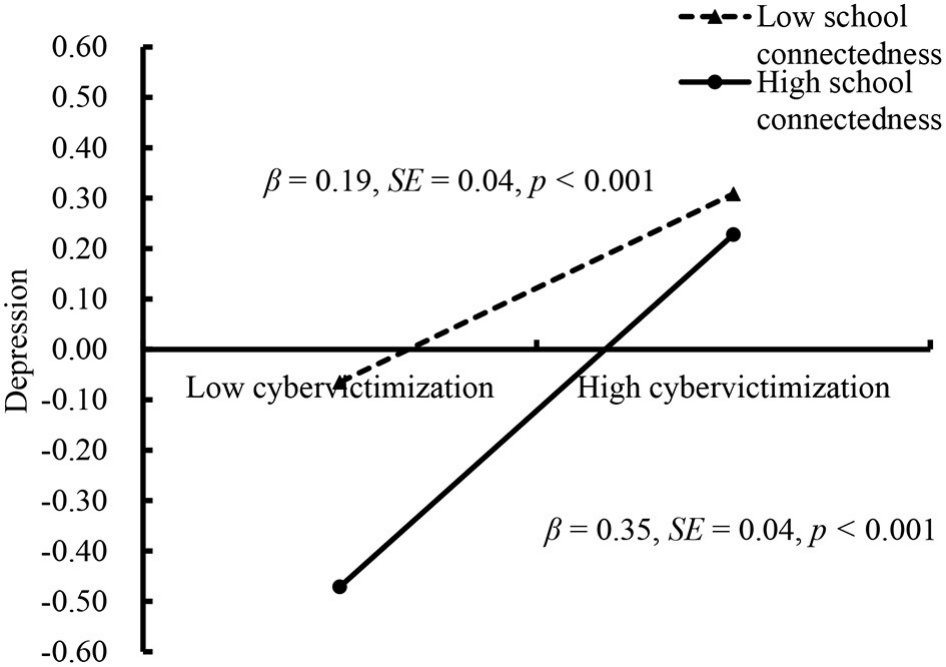
Depression among adolescents as a function of cybervictimization and school connectedness.

Because school connectedness significantly moderated the association between cybervictimization and depression, we conducted a simple slopes test to interpret the interaction. The results are depicted in Figure 4. The positive association between cybervictimization and depression was stronger for adolescents with low school connectedness (1 *SD* above the mean; β = 0.35, SE = 0.04, *t* = 8.30, *p* < 0.001, 95% CI [0.27, 0.43]) than for adolescents with low school connectedness (1 *SD* below the mean; β = 0.19, SE = 0.04, *t* = 4.35, *p* < 0.001, 95% CI [0.10, 0.27]).

Finally, the bias-corrected percentile bootstrap results indicated that the indirect link between cybervictimization and NSSI *via* depression was stronger for adolescents with high school connectedness (indirect effect = 0.12, SE = 0.03, 95% CI [0.07, 0.18]) than for adolescents with low school connectedness (indirect effect = 0.06, SE = 0.02, 95% CI [0.03, 0.11]). Therefore, the mediating effect of depression in the association between cybervictimization and adolescent NSSI was moderated by school connectedness.

## 4. Discussion

NSSI has grown in importance as a problem for adolescent mental health, and according to current study, cybervictimization is a risk factor for NSSI and cybervictimization is a predictor of depression, which is strongly linked to NSSI. Our findings supported the hypothesized model in which depression mediates the association between cybervictimization and NSSI, and this association is weakened by school connectedness. The results of this study offer empirical evidence in support of models on emotion regulation and interpersonal relationships (1, 54).

There was a substantial positive predictive association between cybervictimization and NSSI after adjusting for gender, age, and sensation seeking. The more that adolescents experienced cybervictimization, the more likely they were to show NSSI. This finding is consistent with previous studies (23, 55, 56) and interpersonal model of NSSI (54). Adolescents who experience cybervictimization will experience higher interpersonal pressure, and they may turn to NSSI more frequently as a coping mechanism (56, 57).

Depression played a mediating role in the association between cybervictimization and NSSI, supporting Hypothesis 2. Cybervictimization, depression, and NSSI have been shown to be intercorrelated in prior research. (58–60), but few studies have tested a conceptual model that would explain these relationships. However, because of the fast development of network technology, interpersonal conflicts on the network are not limited by space or time, which might be more harmful to adolescents' mental health (61). Adolescents who experience cybervictimization, as an example of poor interpersonal relationships, may be at risk for depressive symptoms (62, 63). Adolescents may use NSSI to avoid experiencing such negative emotions (27, 56), in accordance with the NSSI's emotion regulation theory (1).

Further research has shown that school connectedness might moderate the association between cybervictimization and depression (64). At the same time, the interaction effect between cybervictimization and connectedness further moderated the relationship between cybervictimization and NSSI. There was an interaction between cybervictimization and school connectedness in predicting adolescents' depression. In those with low school connectedness, cybervictimization may increase symptoms of depression (34). High School connectedness may provide more support to adolescents for their mental health (65, 66). High school connectedness can provide students with a better interpersonal atmosphere in the school environment, thereby lowering the negative impacts of cybervictimization and the risk of NSSI.

This study is the first to document an influence of social support in the form of school connectedness as a protective factor in the association between cybervictimization and NSSI. It was also found that depression may have a mediating effect in the relationship between cybervictimization and NSSI. Based on the results of this study, we propose a Comprehensive Social Support and Emotion Model to explain the link between cybervictimization and NSSI. People who are impacted by negative interpersonal interactions are more prone to experience negative emotions such as depression and anxiety, and NSSI is provides a way to cope with those feelings (54). In this process, social support may play a moderating role, and support from different interpersonal relationships can regulate and alleviate previous interpersonal relationship problems (38).

However, there are still limitations of this study that will need to be addressed in further research. First, the participants were junior high school students from China's Guangdong Province. Despite the homogeneity of the sample, the findings may not adequately account for potential differences, such as geographical differences, racial differences and so on. Second, all of the data used in this study were self-reported. Although the students' responses were anonymous, social desirability may have had some impact, like they will unreal presentation their NSSI situation. In addition, emotional problems such as depression may not be easily observed and assessed by others, and NSSI behaviors usually occur in secret. Therefore, future research can use more sophisticated assessment techniques, such as ecological momentary assessment (EMA) and evaluation from a variety of perspectives (teachers, parents, and important friends). Third, the majority of the data in this study were collected using a cross-sectional design, which can establish association but not causality. Longitudinal research would allow stronger inferences about causality. Fourth, we did not consider the moderating effect of school connectedness on the relationship between depression and NSSI in this study, and future studies may need to consider the moderating effect of school connectedness in a comprehensive manner. Finally, the proposed interpersonal emotion integrated model of NSSI needs additional empirical support. In the future, we also need to conduct a longitudinal study on this issue to further explore the bidirectional relationship and causality between cybervictimization and NSSI.

The findings of this study add to our understanding of the development of NSSI, emphasize the significance of negative emotions and social support in the relationship between cybervictimization and NSSI, offer new perspectives for subsequent research, and have implications for the prevention and intervention of NSSI in adolescents.

## Data availability statement

The raw data supporting the conclusions of this article will be made available by the authors, without undue reservation.

## Ethics statement

The studies involving human participants were reviewed and approved by the Academic Ethics Review Committee of the School of Education, Guangzhou University. Written informed consent to participate in this study was provided by the participants' legal guardian/next of kin.

## Author contributions

Conceptualization and writing-original draft preparation: SL, HZ, WW, and CY. Methodology: CY and HZ. Software: SL, WW, and HZ. Validation: HZ, CY, SZ, and WW. Formal analysis and visualization: CY, HZ, and WW. Investigation: SZ, SL, WZ, and LX. Resources: CY. Data curation and supervision: CY and SZ. Writing—review and editing: HZ, WW, SZ, and SL. Project administration: CY, WZ, and SZ. All authors have read and agreed to the published version of the manuscript.
